# Comparison of Two Commercial Automated Nucleic Acid Extraction and Integrated Quantitation Real-Time PCR Platforms for the Detection of Cytomegalovirus in Plasma

**DOI:** 10.1371/journal.pone.0160493

**Published:** 2016-08-05

**Authors:** Huey-Pin Tsai, You-Yuan Tsai, I-Ting Lin, Pin-Hwa Kuo, Tsai-Yun Chen, Kung-Chao Chang, Jen-Ren Wang

**Affiliations:** 1 Department of Pathology, National Cheng Kung University Hospital, Tainan, Taiwan; 2 Department of Internal Medicine, National Cheng Kung University Hospital, Tainan, Taiwan; 3 Medical Laboratory Science and Biotechnology, National Cheng Kung University, Tainan, Taiwan; 4 Center of Infectious Disease and Signaling Research, National Cheng Kung University, Tainan, Taiwan; 5 National Institute of Infectious Diseases and Vaccinology, National Health Research Institutes, Tainan, Taiwan; University of Kentucky, UNITED STATES

## Abstract

Quantitation of cytomegalovirus (CMV) viral load in the transplant patients has become a standard practice for monitoring the response to antiviral therapy. The cut-off values of CMV viral load assays for preemptive therapy are different due to the various assay designs employed. To establish a sensitive and reliable diagnostic assay for preemptive therapy of CMV infection, two commercial automated platforms including *m*2000sp extraction system integrated the Abbott RealTime (*m*2000rt) and the Roche COBAS AmpliPrep for extraction integrated COBAS Taqman (CAP/CTM) were evaluated using WHO international CMV standards and 110 plasma specimens from transplant patients. The performance characteristics, correlation, and workflow of the two platforms were investigated. The Abbott RealTime assay correlated well with the Roche CAP/CTM assay (R^2^ = 0.9379, P<0.01). The Abbott RealTime assay exhibited higher sensitivity for the detection of CMV viral load, and viral load values measured with Abbott RealTime assay were on average 0.76 log_10_ IU/mL higher than those measured with the Roche CAP/CTM assay (P<0.0001). Workflow analysis on a small batch size at one time, using the Roche CAP/CTM platform had a shorter hands-on time than the Abbott RealTime platform. In conclusion, these two assays can provide reliable data for different purpose in a clinical virology laboratory setting.

## Introduction

Human cytomegalovirus (CMV) is a double-stranded DNA virus that belongs to the β-herpesvirus subgroup of the human herpesvirus family. After primary infection, CMV establishes life-long latency. CMV infections could be asymptomatic, and mild or subclinical diseases in immunocompetent individuals. However, it may lead to severe life-threatening CMV diseases including pneumonia, gastrointestinal disease, hepatitis, CNS disease, retinitis, nephritis, myocarditis in immunocompromised individuals (such as transplant recipients and AIDS patients) [1]. In bone marrow and hematopoietic stem cell transplant (BMT and HSCT) recipients, reactivation of CMV can cause significant morbidity and mortality. Reactivation of CMV by day 100 after BMT occurs in near 80% of CMV-seropositive recipients, and in 28% of seronegative recipients who receive a graft from a seropositive donor [2–4].

Ganciclovir or valacyclovir can be used for prevention of CMV disease and antiviral prophylaxis of CMV infection. Effective therapy for all recipients at risk of CMV reactivation and preemptive therapy for patients with proven CMV reactivation have reduced both morbidity and mortality [5, 6]. Therefore, the use of sensitive and reliable diagnostic assay plays an important role in the preemptive treatment of CMV infection in the early post-transplant period [2, 7]. CMV pp65 antigenemia assay, commercial (*In vitro* diagnostic, IVD) and in-house (laboratory-developed) viral load (real-time polymerase chain reaction) assays were commonly used to monitor CMV activity in transplant patients [2, 7]. In the 1990s, the antigenemia assay was the guidance for preemptive therapy in transplant patients [8]. Subsequently, CMV viral load assays overcame the limitation of operator-dependent variability in the antigenemia assay. In addition, current viral load assays have better precision, broader linear range, faster turnaround time, higher throughput, and less risk of contamination compared with conventional polymerase chain reaction tests. [9, 10]. The test for CMV viral load requires expensive equipment and reagents, although it is less complex with the availability of an increasing number of commercial reagents.

CMV reactivation occurred in 51% and 41.8% of the BMT recipients as detected by in-house real-time PCR and antigenemia assays in peripheral blood leukocytes, respectively [4]. CMV DNA cut-off values in both plasma and whole blood have been proposed for preemptive treatment [11–13]. However, the cut-off values of viral load assays for preemptive therapy are different due to the various assay designs employed and the lack of an international reference standard [14, 15]. Since 2010, the World Health Organization (WHO) International Standard from the National Institute of Biological Standards and Controls (United Kingdom) has been used for converting values between assays which are reported in IU/mL. However, there remains a considerable variability of extraction methods and quantitative real-time PCR methods in the laboratory [16, 17]. In addition, the results of intra- and inter-laboratory variability, limit of detection (LOD), and precision of assays may impact comparability and clinical practice [18, 19]. The cutoff value and interpretation of results must depend on the assay, specimen type, degree of immune suppression, type of transplant, and CMV immune status of the donor/recipient. An accurate starting viral load is also critical when monitoring the results in the same patient [20]. Thus, it is very important to verify the values obtained using various assays in the detection of CMV viral load. The goal of this study was to evaluate CMV viral load in two automated nucleic acid extraction platforms and integrated real-time PCR based systems, the Abbott RealTime (ART) CMV on the m2000 system with *in vitro* diagnostic (IVD) Conformité Européene (CE)-labeled certification and the Food and Drug Administration (FDA)-approved Roche COBAS AmpliPrep/COBAS Taqman (CAP/CTM) CMV assay, in order to have a sensitive and reliable viral load assay for treatment.

## Materials and Methods

### CMV WHO international standard

The WHO international standard (IS) obtained from the National Institute for Biological Standards and Control (NIBSC code: 09/162, Great Britain) for viral load assays is a lyophilized whole-virus preparation of the CMV Merlin strain. After reconstitution in 1 mL of water, the WHO IS stock has a concentration of 5x10^6^ IU/mL (i.e. 6.7 log_10_ IU/mL).

### Analytical performance of the viral load assays

The WHO IS stock was diluted 10-fold serially to obtain a 5-member panel with concentrations at 5x10^5^(A), 5x10^4^(B), 5x10^3^(C), 5x10^2^(D) and 50 IU/ml (E). Each dilution sample was performed by Abbott RealTime (RT) CMV assay and Roche Cobas AmpliPrep/CoBAS TaqMan (CAP/CTM) CMV assay. This panel was used to verify precision, accuracy, and linearity of the assays.

### Automated CMV viral load assays

The Roche CMV assay runs on the CAP/CTM system (Roche Molecular Diagnostics, Pleasanton, CA, USA) which consists of the COBAS AmpliPrep for sample preparation and the COBAS Taqman for realtime PCR. The Roche CAP/CTM CMV test uses primers and probes targeting a conserved region of the CMV genome (UL54, encodes DNA polymerase). This assay uses an automatic, magnetic bead nucleic acid isolation using the COBAS AmpliPrep system. The quantification linear range is from 137 to 9,100,000 IU/mL. The Abbott RealTime CMV assay (Abbott Molecular Inc., Des Plaines, IL, USA), sample preparation was carried out on *m*2000sp using the magnetic bead m2000 System DNA extraction kit, and amplification and detection of the UL34 and UL80.5 genes of CMV were conducted on the *m*2000rt using RealTime CMV kits. The quantification linear range of plasma is from 31 to 156,000,000 IU/mL using the Abbott RealTime CMV assay. Both assays were performed following the instructions of the respective manufacturers.

### Clinical specimens

From Jan 2013 to Jan 2015, a total of 110 plasma specimens from BMT, HSCT patients at National Cheng Kung University Hospital were included in the study. Each plasma specimen was tested by two automated CMV viral load assays

### Ethics statement

Institutional Review Board (IRB) approval of this study was obtained from National Cheng Kung University Hospital (No. B-ER-103-227). This study was retrospective design and conducted in Tainan, Taiwan only. Each patient agreed to the study and signed the informed consent form.

### Statistical analysis

The data were log_10_ transformed prior to analysis. Bias was estimated by the difference between the measured and standard expected values. Any quantitative correlations between the CMV viral loads measured using these two assays were analyzed using Pearson’s correlation tests. The method of Bland and Altman was used to assess the agreement between viral loads measured using MedCalc statistical software.

## Results

### Comparison of analytical performance of the Abbott RealTime CMV and Roche CAP/CTM CMV assays for testing of WHO panels

The reproducibility of between-run and with-run, linearity, and accuracy of the Abbott RealTime CMV and Roche CAP/CTM CMV assays were determined using dilutions of WHO panels. Within-run samples were tested in triplicate on the same run. Positive values from two additional runs were used for determination of between-run precision which was represented by coefficient of variation (CV). The CVs of within-run precision of Abbott RealTime CMV assay and Roche CAP/CTM CMV assay were 0.2–3.10% and 1.57–6.59% (Table 1), respectively. In addition, the precision values of between-run were 0.76–0.78% and 3.01–3.91% for the Abbott RealTime CMV assay and Roche CAP/CTM CMV assay (Table 1), respectively.

**Table 1:** Comparison of analytical performance of Abbott RealTime CMV and Roche CAP/CTM CMV viral load assays.

WHO Panel		Abbott RealTime CMV			Roche CAP/CTM CMV		
Expected values		Mean	SD	CV	Mean	SD	CV
(log_10_ IU/mL)		(log_10_ IU/mL)	(log_10_ IU/mL)	(%)	(log_10_ IU/mL)	(log_10_ IU/mL)	(%)
Within-Run Precision							
A	5.699	5.84	0.01	0.20	5.52	0.16	2.91
B	4.699	4.88	0.02	0.31	4.63	0.30	6.59
C	3.699	3.84	0.03	0.75	3.74	0.06	1.57
D	2.699	2.91	0.03	0.91	2.74	0.12	4.28
E	1.699	1.94	0.06	3.10	<LOD*		
Between-Run Precision							
B	4.699	4.86	0.04	0.76	4.84	0.19	3.91
D	2.699	2.88	0.02	0.78	2.74	0.08	3.01

*LOD: limit of detection

Low viral load values are common and their clinical significance can be difficult to assess. An accurate starting viral load is critical when monitoring response to therapy. Linearity and accuracy were evaluated by testing the WHO CMV standard dilution panel. Three replicates at each concentration level were tested for both the Abbott RealTime and Roche CAP/CTM CMV assay. Both assays demonstrated excellent linearity (R^2^>0.99) (data not shown). The stock WHO standard was also run in single replicate for assessment of accuracy. The viral load results generated using Abbott RealTime CMV and Roche CAP/CTM CMV assay were compared with the expected value in log_10_ IU/ml. The accuracy data showed the bias values of Abbott RealTime CMV assay and Roche CAP/CTM CMV assay were 0.141~0.241 and 0.041~-0.419 (Table 2), respectively.

**Table 2:** Comparison of Accuracy of Abbott RealTime CMV and Roche CAP/CTM CMV viral load assays.

WHO Panel		Abbott RealTime CMV	Bias	Roche CAP/CTM CMV	Bias
Expected values		Mean	(Abbott-Expected)	Mean	(Roche-Expected)
(log_10_ IU/mL)		(log_10_ IU/mL)	(log_10_ IU/mL)	(log_10_ IU/mL)	(log_10_ IU/mL)
	6.699	6.9	0.201	6.28	-0.419
A	5.699	5.84	0.141	5.52	-0.179
B	4.699	4.88	0.181	4.63	-0.069
C	3.699	3.84	0.141	3.74	0.041
D	2.699	2.91	0.211	2.74	0.041
E	1.699	1.94	0.241	<LOD*	ND

*LOD: limit of detection

### Comparison of the performance for clinical specimens by the Abbott RealTime CMV and Roche CAP/CTM CMV assays

**Fig 1:**
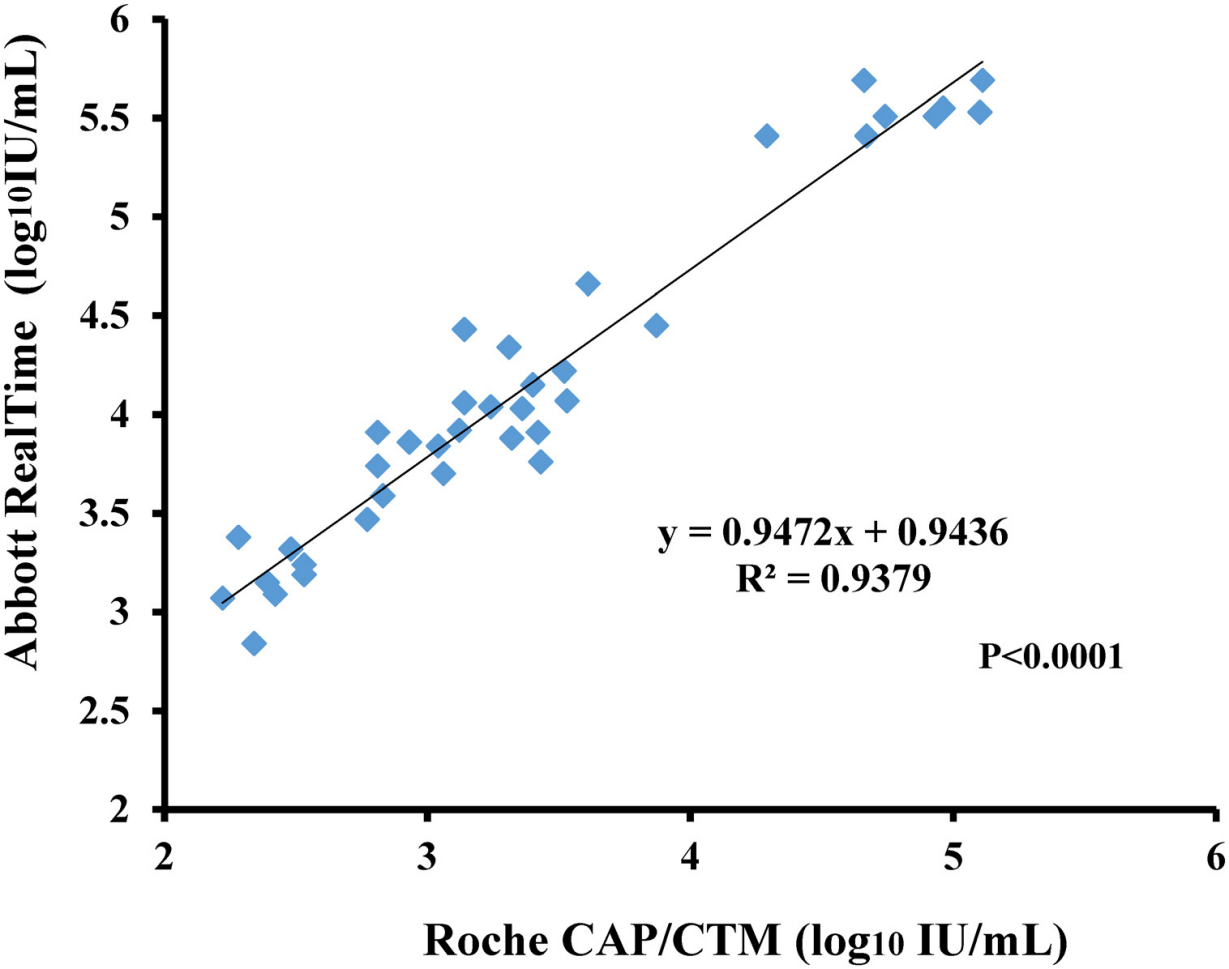
Correlation of CMV viral load (log_10_ IU/mL) in the Abbott RealTime assay and the Roche CAP/CTM assay. All plasma specimens tested positive were included. The data was shown that there was good correlation (R^2^ = 0.9379, P<0.01) in these two platforms.

**Fig 2:**
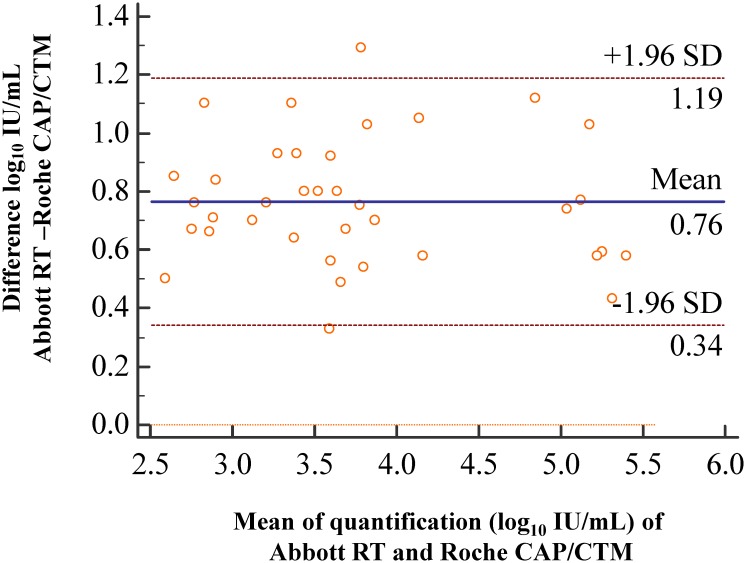
Bland-Altman analysis of the Abbott RealTime CMV and the Roche CAP/CTM CMV assays. Thirth-seven specimens with CMV viral loads (log_10_ IU/mL) above the lower limit of quantification (LLQ) were included. The viral load values of Abbott RealTime assay were higher than the values of the Roche CAP/CTM assay on average 0.76 log_10_ IU/mL (P<0.0001).

One hundred and ten clinical plasma specimens were tested by both assays. The results of qualitative correlation showed that there were 53 concordant positive (38 quantifiable in both assays), 52 concordant negative, and 5 discordant specimens (Table 3). Fifty-four quantifiable specimens using the Abbott RealTime assay showed 1 negative and 15 below LOD results using the Roche CAP/CTM assay, and 4 specimens below LOD results using the Abbott RealTime CMV assay showed negative results using the Roche CAP/CTM CMV assay. Using a true positive defined as one positive assay for either of the assays as the gold standard, the sensitivity and specificity of the assays were 100% and 100% for Abbott RealTime CMV assay, and 91.3% and 100% for Roche CAP/CTM CMV assay, respectively. For the 38 positive specimens quantified by both assays, there was one discordant sample observed between two assays which value of Abbott RealTime CMV assay was about 0.74 log_10_ lower than the Roche CAP/CTM CMV assay result. This sample was subsequently tested on Roche CAP/CTM CMV assay and viral load quantitation was still higher than the origin Abbott RealTime CMV assay data upon repeat testing. No further investigation was performed for Abbott assay on this sample due to out of the sample (S1 File). The CMV viral load data of both assays (N = 37) were significantly correlated (R^2^ = 0.9379, P<0.001) (Fig 1). Bland-Altman analysis showed that viral load values measured with Abbott RealTime CMV assay were on average of 0.76 log_10_ IU/mL higher than those measured with the Roche CAP/CTM CMV assay (P<0.0001) and the difference in viral load based on the 95% confidence interval (CI) was 0.6930–0.8367 log_10_ IU/mL (Fig 2). At the upper end of the dynamic range (> 4.0 log_10_ IU/mL in Roche), we observed a difference of 0.73 log_10_ IU/mL (N = 8) between Abbott RealTime CMV and Roche CAP/CTM CMV assays while at the lower end (2.2–2.5 log_10_ IU/mL in Roche) of the dynamic range a difference of 0.76 log_10_ IU/mL (N = 8) was observed among these positive specimens.

**Table 3:** Qualitative correlation in clinical specimens on Abbott RealTime CMV and Roche CAP/CTM CMV viral load assays.

		**Roche CAP/CTM**		
N = 110		Not Detected	Detected, but< LOQ	Quantifiable
	Not Detected	52	0	0
**Abbott RealTime**	Detected, but< LOQ*	4	0	0
	Quantifiable	1	15	38

*LOQ: lower limit of quantification

### Comparison of the Workflow on the Abbott RealTime CMV and Roche CAP/CTM CMV platform

**Fig 3:**
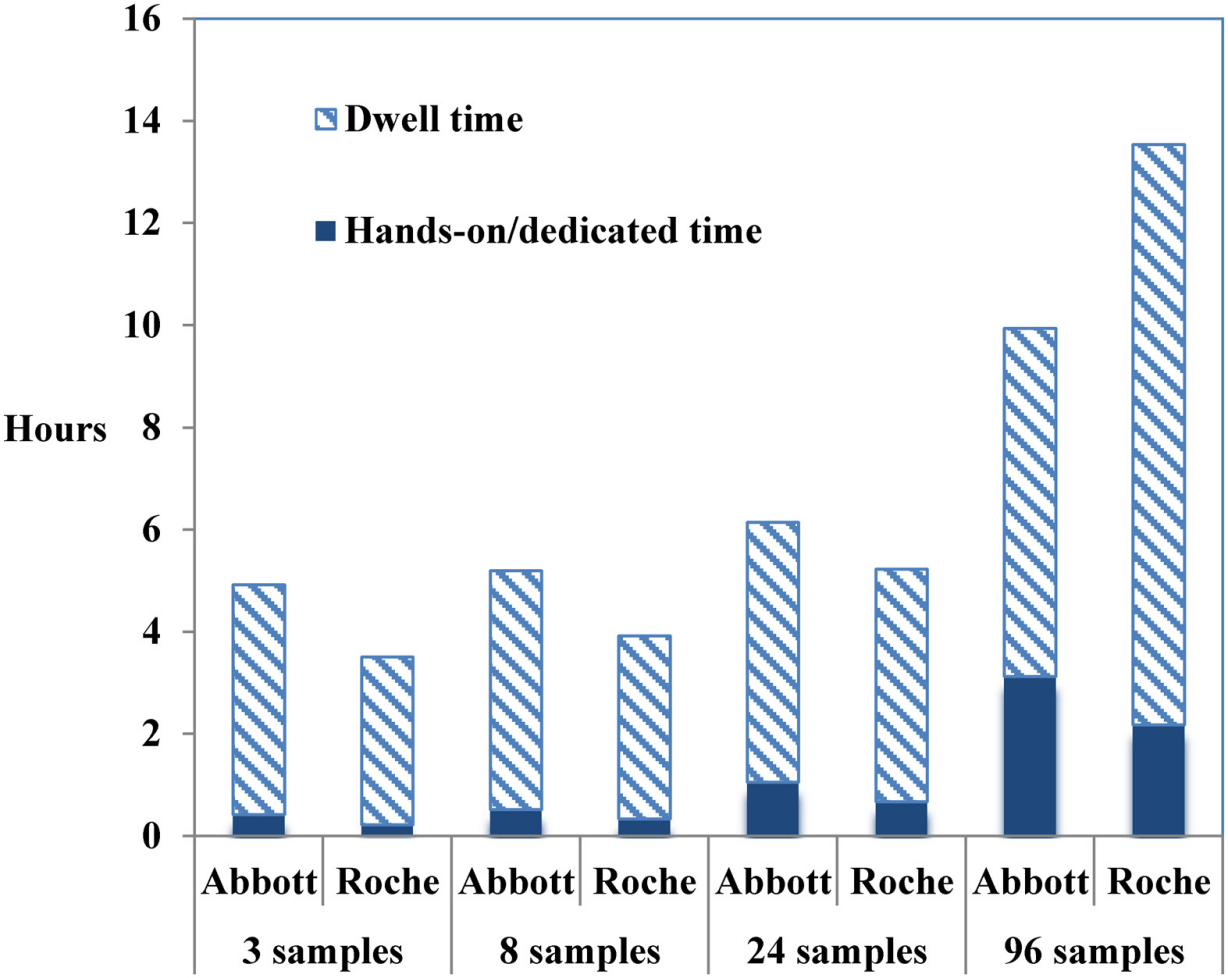
Comparative workflow analysis between the Abbott RealTime CMV and the Roche CAP/CTM CMV platforms. Total turnaround time was further broken down into hands-on time and dwell time. Dwell time means time spent in the same position, area and stage of a process. The data showed that it is more convenient to user for operating in the Roche CAP/CTM CMV platform.

Workflow analysis is critical in the diagnostic laboratory. A workflow study was carried out to compare the turnaround time and hands-on time for different sample batch sizes. We found that with the exception of the 96-sample batch size, a 24-sample or less than 24-sample batch size had a shorter turn-around time using the Roche CAP/CTM CMV platform compared with that for the Abbott RealTime CMV platform. The workflow analysis revealed the procedures performed using the Roche CAP/CTM CMV platform had a shorter hands-on time than that of the Abbott RealTime CMV platform (Fig 3).

## Discussion

Quantitation of cytomegalovirus (CMV) viral load in the transplant patients has become a standard practice for monitoring the response to antiviral therapy. However, agreement of quantitative values among different platforms is important for monitoring the results of the same patient obtained from different institutions or periods. In this study, our data showed that Roche CAP/CTM CMV had larger CV values than Abbott RealTime CMV assay in within-run and between-run precision results when using the WHO IS dilutions. Larger bias was observed for the high WHO IS concentration in the Roche CAP/CTM CMV assay and for the low WHO IS concentration in the Abbott RealTime CMV assay. Based on the data in Table 1, the extraction/PCR results for the WHO panel is not linear for the Roche CAP/CTM CMV assay (data not shown). Although both assays demonstrated excellent linearity, the Roche CAP/CTM CMV assay underestimated viral loads at the upper end of the dynamic range while the quantitation of viral loads in the middle of the dynamic range was in line with the expected values. This difference in quantitation across the dynamic range could be due to the fact that Roche CAP/CTM CMV assays use an internal quantitation standard (IQS) to calibrate viral load results. This single IQS may not be able to adjust for variances at both the upper and lower end of the dynamic range. Number of hepatitis C studies have demonstrated that Roche CAP/CTM results indeed are not linear across the entire dynamic range and require the use of a third order polynomial linear regression with ±0.2 log10 IU/ml allowable maximum difference from linearity [21, 22]. In contrast, the Abbott RealTime assay employs a simple linear function using a two point external calibration strategy to quantitate viral load results [23]. This application of the external calibration strategy has demonstrated that Abbott RealTime assays are linear across the entire dynamic range. Hence, it is possible that a contributing factor to the observed bias across the dynamic range is the calibration strategy utilized by each assay.

The sensitivity (100%) of the Abbott RealTime CMV assay was higher than that of the Roche CAP/CTM CMV (91.3%) for the detection of CMV in clinical specimens. Other reports also showed that the Abbott RealTime CMV assay has good sensitivity, precision, and linearity when compared to the Roche Cobas Amplicor CMV assay (PCR-ELISA) and other in-house assays [24, 25]. According to the manufacturers' instructions for the Abbott RealTime CMV and the Roche CAP/CTM CMV assays, the design of the calibrator and control (Abbott RealTime CMV assay with external control as calibrator and Roche CAP/CTM CMV assay with internal control as quantitation standard) may affect the performance of precision, sensitivity, and linearity in both systems.

In the Bland Altman analysis, our data were similar to those reported by Clari et al. [26], which showed viral loads of the Abbott RealTime CMV PCR were on average approximately 1.0 log_10_ IU/mL higher than those of the Abbott CMV PCR kit. In a study by BaBady et al., whole blood and plasma by the Roche CAP/CTM CMV assay and the Roche CMV analyte-specific reagent on the LightCycler 2.0 (LC) with plasma were compared to monitor CMV viral loads. However, their results showed that the plasma sample in the Roche CAP/CTM CMV assay had the highest sensitivity and lowest specificity for the detection of CMV. In addition, the viral load with whole blood for individuals was on average 0.5- to 1.22-log higher than those in plasma [27]. Plasma and whole-blood specimens both provide prognostic and diagnostic information regarding CMV. CMV DNA is generally detected earlier and in greater quantitative amounts (about 10-fold higher) in whole blood compared with plasma [28–31]. Due to latent DNA persists in the cellular fraction of blood, the detection of CMV in plasma is considered more likely to reflect active infection. Thus, in this study, the viral loads detected with the Abbott RealTime CMV assay may reveal an actual result to therapy response.

Low levels of CMV DNA in whole blood or plasma (<100–500 copies/mL) may not correlate with the development of disease. However, a viral load value of 2000–5000 copies/mL correlated with the development of end-organ disease using quantitative polymerase chain reaction (PCR) [32,33]. Moreover, the increased sensitivity of the CMV viral load assay and the low-level viremia detection may prolong therapy and increase toxicity to patients. Thus, additional studies are needed to better understand clinical applications of the improved sensitivity with the CMV DNA assays.

There are more manual intervention procedures in the Abbott RealTime CMV platform than in the Roche CAP/CTM CMV platform such as sample preparation and PCR setup. Thus, the Roche CAP/CTM CMV platform with its short hands-on operating time was more convenient than the Abbott RealTime CMV platform for small batch size specimens (24 or less) in the laboratory workflow analysis. However, due to the limited sample number (maximum 24 in the Roche CAP/CTM CMV platform, 48 in the Abbott RealTime CMV platform) that can be accessed at one time, the turnaround time of the Abbott RealTime CMV assay is shorter than that of the Roche CAP/CTM CMV assay for large batch size specimens. Overall, these two systems provided reliable and comparable results. Although the performance characteristics of the Abbott RealTime CMV assay were better than those of the Roche CAP/CTM CMV assay, the Roche CAP/CTM CMV assay provides rapid test results for batch sizes of less than 24 samples. In conclusion, these data may be helpful in the selection of a suitable platform for different purpose in the clinical virology laboratory.

## Supporting Information

S1 File Dataset and Sources. An excel file contained the data used in this study.
(XLSX)
